# The Effect of Amino Acids on Wound Healing: A Systematic Review and Meta-Analysis on Arginine and Glutamine

**DOI:** 10.3390/nu13082498

**Published:** 2021-07-22

**Authors:** Elena Arribas-López, Nazanin Zand, Omorogieva Ojo, Martin John Snowden, Tony Kochhar

**Affiliations:** 1 School of Science, Medway Campus, University of Greenwich, Central Ave, Gillingham, Chatham Maritime, Kent ME4 4TB, UK; E.ArribasLopez@greenwich.ac.uk (E.A.-L.); M.J.Snowden@greenwich.ac.uk (M.J.S.); 2 School of Health Sciences, Avery Hill Campus, University of Greenwich, Avery Hill Road, London SE9 2UG, UK; O.Ojo@greenwich.ac.uk; 3 HCA London Bridge Hospital, Tooley Street, London SE1 2PR, UK; tonykochhar@gmail.com

**Keywords:** arginine, collagen deposition, contraction, food, glutamine, growth factor, gut permeability, interleukin, re-epithelialization, wound healing

## Abstract

Under stress conditions, the metabolic demand for nutrients increases, which, if not met, may slow down or indeed stop the wound from healing, thus, becoming chronic wounds. This study aims to perform a systematic review and meta-analysis of the effect of arginine and glutamine supplementation on wound healing. PRISMA (Preferred Reporting Items for Systematic Reviews and Meta-Analyses) guidelines were followed for the systematic review and ten electronic databases were used. Five and 39 human studies met the inclusion criteria for arginine and glutamine, respectively. The overall meta-analysis demonstrated a significant effect of arginine supplementation on hydroxyproline content (MD: 4.49, 95% CI: 3.54, 4.45, *p* < 0.00001). Regarding glutamine supplementation, there was significant effect on nitrogen balance levels (MD: 0.39, 95% CI: 0.21, 0.58, *p* < 0.0001), IL-6 levels (MD: −5.78, 95% CI: −8.71, −2.86, *p* = 0.0001), TNFα levels (MD: −8.15, 95% CI: −9.34, −6.96, *p* < 0.00001), lactulose/mannitol (L/M) ratio (MD: −0.01, 95% CI: −0.02, −0.01, *p* < 0.00001), patient mortality (OR: 0.48, 95% CI: 0.32, 0.72, *p* = 0.0004), C-reactive protein (CRP) levels (MD: −1.10, 95% CI: −1.26, −0.93, *p* < 0.00001) and length of hospital stay (LOS) (MD: −2.65, 95% CI: −3.10, −2.21, *p* < 0.00001). Regarding T-cell lymphocytes, a slight decrease was observed, although it failed to reach significance (MD: −0.16, 95% CI: −0.33, 0.01, *p* = 0.07). Conclusion: The wound healing might be enhanced in one or at various stages by nutritional supplementation in the right dose.

## 1. Introduction

**Figure 1:**
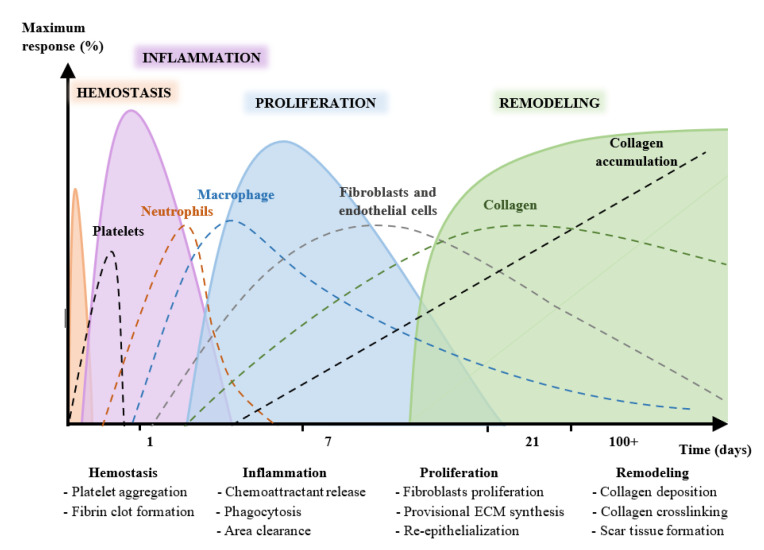
Stages of skin wound healing (hemostasis, inflammation, proliferation, and repair and remodeling) over time.

A wound is known as the disruption in the physical continuity of functional tissues [1,2,3,4]. The healing process begins immediately after an injury [5,6,7,8] and involves four phases [3,9,10,11,12,13]. The healing process consists of a series of sequential and overlapping physiological phases or stages that can persist for years [9,14,15,16,17], as shown in Figure 1. It is not a linear process and, depending on diverse extrinsic and intrinsic factors, such as growth factors and cytokines, it can progress both backward and forward through the stages.

### 1.1. Nutrition

Nutrition is recognized as a key factor in wound healing. Under conditions of stress such as trauma or after surgery, the nutritional demand is increased [18,19,20] in part due to cell proliferation and protein synthesis [21].

**Figure 2:**
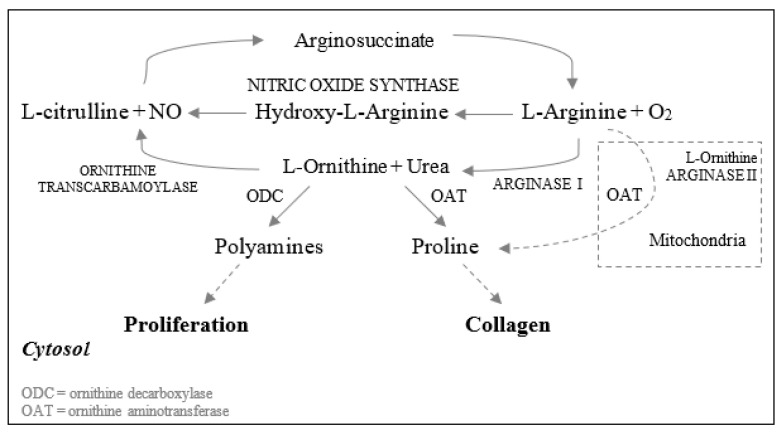
Metabolism of L-Arginine to produce NO and metabolites involved in the wound healing process. Arginine can be catabolized via the NO synthase pathway. Here, L-Arginine can be converted to L-ornithine and urea by arginase I. Then, by the action of ornithine aminotransferase, ornithine is transformed into proline, which is needed for collagen synthesis. L-ornithine can also be converted to polyamines, which are required for cell proliferation by ornithine decarboxylase [22].

Arginine is a conditionally essential amino acid that is synthesized from citrulline in healthy humans [22] (Figure 2**)**. Based on previous reviews, arginine has been shown to modulate the immune function, hormone secretion, and endothelial function as well as being a precursor to the synthesis of proline in animal and human trials [23,24,25].

There are two pathways in wound healing involving arginine (a) the arginase pathway, which produces polyamines, as well as ornithine and proline. Polyamines are needed for cell proliferation, while the latter ones are required for the synthesis of collagen; and (b) the inducible nitric oxide (NO) synthetase or iNOS pathway, which is a precursor of nitric oxide (Figure 2). NO plays a key role in wound healing as it regulates cell proliferation, collagen formation, and wound contraction [22,26].

Glutamine is the most abundant amino acid found in human blood plasma. It is used as a source of energy for the cells to proliferate, including lymphocytes, macrophages, fibroblasts, and epithelial cells [21,23]. Similar to arginine, its concentration in plasma decreases under conditions of metabolic stress, such as injury, and its depletion is proportional to the acuteness of the trauma [27,28,29,30].

**Figure 3:**
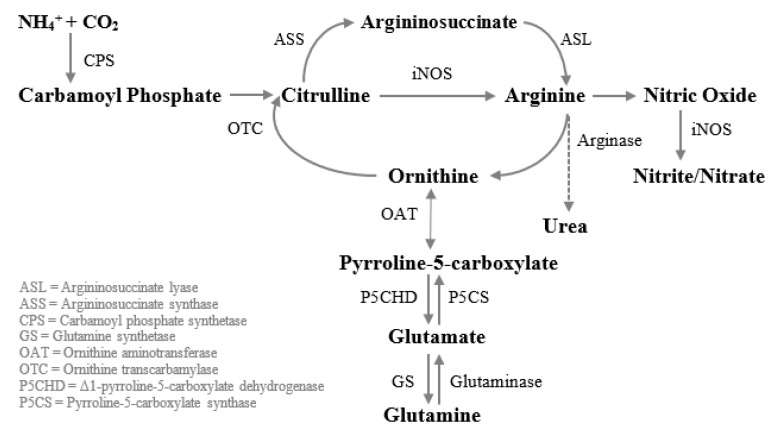
Metabolism of glutamine to arginine in human macrophages. Carbamoyl phosphate when combined with ornithine via OTC is converted to citrulline. Then citrulline is transformed into argininosuccinate and then into arginine by the action of ASS and ASL, respectively. Arginine can then be turned into nitric oxide or ornithine. Ornithine can be transformed into glutamine, and vice versa, via glutamate and pyrroline-5-carboxylate.

Some studies have demonstrated that glutamine enhances wound healing, in part, because it increases the concentration of arginine and citrulline, a precursor of arginine (Figure 3). Glutamine thus allows the production of NO in the absence of extracellular arginine in monocytes and macrophages [28,31,32,33,34]. This amino acid also reduces gut atrophy [35,36,37,38,39] and limits intestinal permeability [40,41,42,43,44,45,46], indirectly reducing the production of proinflammatory cytokines [47]. The intestinal permeability, measured by the lactulose/mannitol excretion ratio, is an important parameter in wound healing since its increment is correlated with the production of proinflammatory cytokines, such as interleukin-6 (IL-6) [48] which plays a key role in the modulation of healing through the regulation of differentiation, activation, and proliferation of keratinocytes, leukocytes, fibroblasts and endothelial cells [49]. Glutamine has also been shown to reduce C-reactive protein (CRP). CRP plays important roles in inflammatory processes and hosts reactions against infections, including NO release, apoptosis, and the production of IL-6 and tumor necrosis factor-α (TNFα). Therefore, CRP levels increase in sick patients and are correlated with the severity of the illness in the patient, thus, objectively quantifying the patient’s stress and acuity [50,51]. Hence, the decline of CRP indicates the reduction of the overall inflammation. Glutamine also acts as an antioxidant through the production of glutathione (GSH) [29,52].

### 1.2. Why It Is Important to Do This Review

Both arginine and glutamine are considered conditionally essential amino acids. Therefore, they are needed in stress conditions and thus, for wound healing to occur. There is existing information suggesting their mechanism of action in the different stages of healing. However, it is our belief that no previous systematic review and meta-analysis has been conducted on arginine supplementation and its effect on wound healing. In the case of glutamine, its beneficial effect on hospital stay has been reported by two meta-analyses performed by Bollhalder et al. [53] and Novak et al. [54]. However, the first review only included enteral supplementation and focused on the length of hospital stay and mortality. Whereas the second one focused on surgery and critically ill patients. To our knowledge, there is currently no evidence of the effect of their supplementation on different variables affecting wound healing.

This systematic review and meta-analysis aimed to evaluate the effect of supplementation of arginine or glutamine on wound healing or parameters related to healing.

## 2. Materials and Methods

The following systematic review was performed according to the Preferred Reporting Items for Systematic Review and Meta-Analysis (PRISMA) statement [55]. Furthermore, this review follows the Population, Intervention, Comparison, and Outcome (PICO) characterization.

### 2.1. Search Strategy

The databases searched for relevant papers published before October 2020 were Pubmed, American Physiological Society Publications, Taylor & Francis Online, Web of Science, EMBASE, grey literature research with Google Scholar.

Based on the search strategy, the following keywords and synonyms/medical subject headings were used: arginine or glutamine and (inflammation or healing or wound or surgery or cytokines or interleukin or nutrition or hospital stay or C-reactive protein). Words were combined using Boolean operators (OR/AND) (Table 1). References from pertinent articles were also examined for additional studies. Searches were conducted and data from the selected articles were extracted by one researcher (E.A.L.) and cross-checked by another researcher (NZ). For the meta-analysis data, the authors of the selected articles were contacted for the original data when needed.

### 2.2. Study Selection

**Figure 4:**
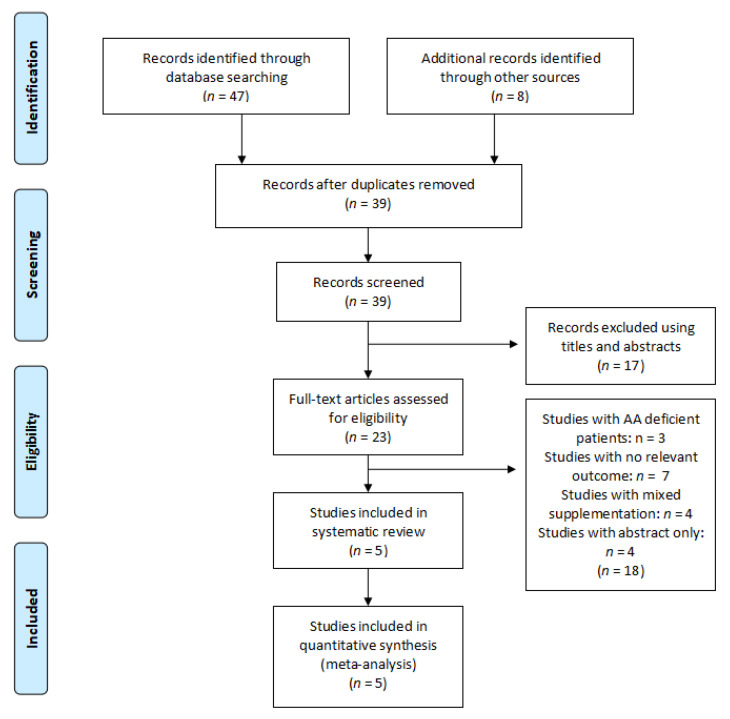
Flow diagram of the search strategy for arginine.

**Figure 5:**
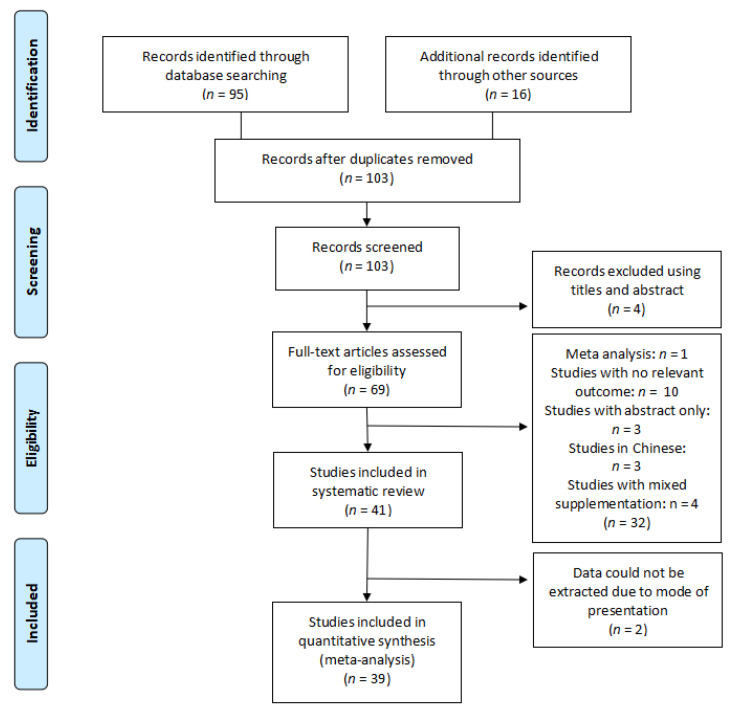
Flow diagram of the search strategy for glutamine.

Inclusion criteria: studies selected were randomised controlled trials (RCTs) where patients above 18 years old, healthy or not, suffering from acute or chronic wounds were supplemented with arginine or glutamine (Figure 4 and Figure 5).

Exclusion criteria: Studies that did not entail in vivo human studies involving supplementation with arginine or glutamine were excluded from the review. Studies involving participants below 18 years of age were excluded from the review due to the metabolic stress already occurring resulting from growth. Studies involving patients with diabetes, where data were not complete or the original data were not presented and studies in another language other than English, Spanish or French were excluded from the review.

#### 2.2.1. Population

Adults above 18 years old, healthy or not, suffering from acute or chronic wounds.

#### 2.2.2. Intervention

Diet supplemented with either arginine or glutamine for at least 5 days.

#### 2.2.3. Comparator

A control group, either treated with a placebo or not treated.

#### 2.2.4. Outcomes

The outcomes included in the meta-analysis were: nitrogen balance (g N), hydroxyproline content (nmol/cm), T-cell lymphocytes levels, tumour necrosis factor-α (TNFα) levels (pg/mL), C-reactive protein (mg/dL), intestinal permeability (L/M ratio), length of hospital stay (LOS) (days), patient mortality (deaths), IL-6 levels (mg/dL).

### 2.3. Data Extraction and Management

Data for the meta-analysis were extracted from figures using WebPlotDigitizer [56], tables and the test from the articles, and the change in mean and standard deviation between the baseline and final values for each outcome were used for the meta-analysis. No publication date restrictions were applied. Units of measurements were converted to mg/dL for CRP as necessary. Additionally, median values were converted to means and 1st–3rd quartiles were transformed into standard deviations, respectively.

### 2.4. Quality Assessment

The risk of bias assessment was assessed by the Cochrane risk of bias tool [57]. The domains evaluated included the random sequence generation (selection bias), allocation concealment (selection bias), blinding of participants and personnel (performance bias), blinding of outcome assessment (detection bias), incomplete outcome data (attrition bias), selective reporting (reporting bias), and other bias. Low risk of bias is indicated by a plus (+), unclear risk of bias by a question mark (?), and high risk of bias by a minus (−).

### 2.5. Data Analysis

Changes from baseline for the intervention were compared with the control in all the parameters analyzed [57]. The pooling of the data was conducted with the meta-analytic methodology, utilizing Cochrane Review Manager 5.4.1 (2020) [58] for the different outcomes evaluated applying fixed effects, the mean differences (MDs) and odds ratio as a degree of effect extent. Nevertheless, for nitrogen balance and T-cell lymphocytes levels, data were converted into standardized mean difference (SMD) owing to the use of different measurement scales. Pooled effect size estimates are presented with their 95% confidence intervals (95% CI). When studies reported multiple results (i.e., multiple-dose), these were included in the meta-analysis as independent comparisons. Heterogeneity was assessed using I^2^ and Chi^2^ and considered significant when I^2^ > 50%. Results were considered significant when the *p*-value was below 0.05.

## 3. Results

Five (5) and 39 studies on arginine and glutamine, respectively were included in the systematic review (Figure 4 and Figure 5) (Table 2 and Table 3).

### 3.1. Assessment of Risk of Bias of Included Studies

#### 3.1.1. Risk of Bias of Included Studies on Arginine

**Figure 6:**
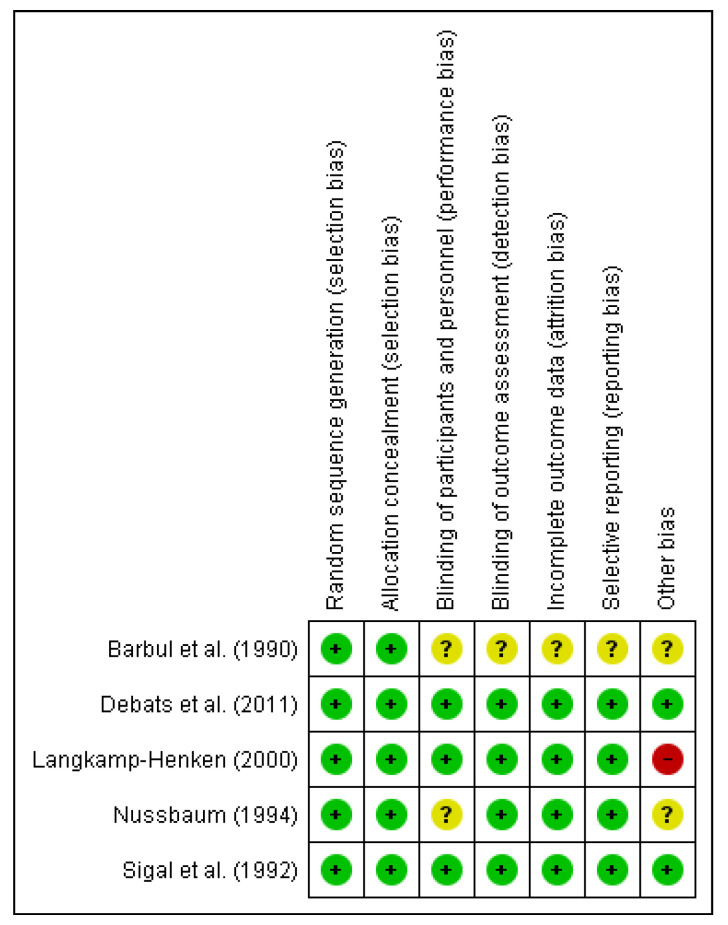
Risk of bias summary for the included studies on arginine. Low risk of bias (+), unclear risk of bias (?), and high risk of bias (−).

The risks of bias in the included studies are shown in Figure 6. 100% of the studies showed a low risk of bias in relation to the random sequence generation and allocation concealment. While less than 75% have demonstrated a low risk of bias with respect to blinding of participants and personnel. In terms of blinding of outcome assessment, incomplete outcome data and selective reporting, all the studies showed a low risk of bias except for Barbul et al. [59] which exhibited an unclear risk of bias. Regarding other risks of bias, less than 50% of the studies showed a low risk of bias whereas the Langkamp-Henken study [60] demonstrated a high risk of bias.

#### 3.1.2. Risk of Bias of Included Studies on Glutamine

**Figure 7:**
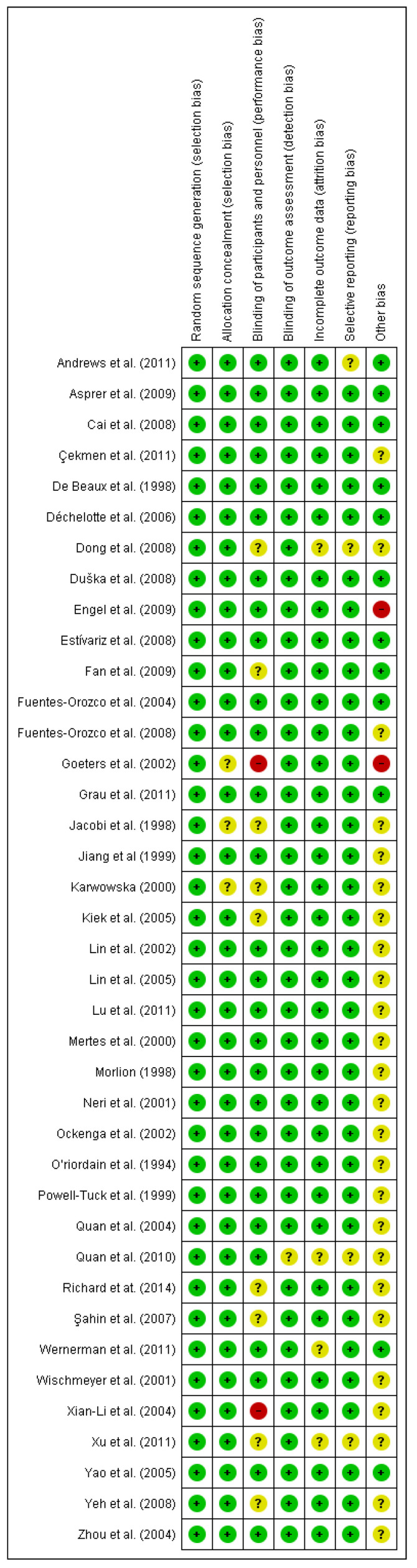
Risk of bias summary for the included studies on glutamine. Low risk of bias (+), unclear risk of bias (?), and high risk of bias (−).

The risks of bias in the included studies are shown in Figure 7. Of the studies, 100% showed a low risk of bias in relation to the random sequence generation. All the studies have demonstrated a low or unclear risk of bias regarding blinding of participants except the Goeters et al. [61] and Xian-Li et al. [62] studies, which exhibited a high risk of bias. On the other hand, more than 50% of the studies showed an unclear and high risk of bias in terms of other biases. The high bias is the case of Engel et al. [63] and Goeters et al. studies [61]. With respect to the other risk of bias, more than 75% of the studies exhibited a low risk of bias regarding allocation concealment, blinding of outcome assessment, incomplete data and selective reporting.

### 3.2. Effects of Interventions

Based on the systematic review and meta-analysis, one distinct area was identified under arginine: Collagen deposition (hydroxyproline content); and seven distinct areas were identified under glutamine: nitrogen balance; wound healing time; length of hospital stay and patient mortality; lactulose/mannitol ratio; C-reactive protein; cytokines (IL-6 levels, TNFα levels) and T-cell lymphocytes.

### 3.3. Arginine (Arg)

Research on the pharmacological effects of arginine supplementation has been mostly based on its use for acute wounds, although some trials have studied its effect on chronic wounds (Table 2).

**Table 2:** Studies evaluating the effect of arginine supplementation on wound healing and reported outcomes.

Study	Duration	Patient Population	*n*	Dosage	Control Group	Outcome
Barbul et al. [59]	14 days	Surgery	36	24.8 g Arg	Not supplemented	↑ Collagen deposition, ↑ Wound-breaking strength, ↑ Lymphocyte mitogenesis
Nussbaum [64]	14 days	Surgery	30	17 g Arg	Not supplemented	↑ Collagen synthesis, ↑ T-cell-mediated immune function, ↑ IGF-1
Debats et al. [65]	5 days	Surgery	35	30 g intravenous Arg	Isonitrogenous solution	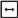 Citrulline, ornithine and NO levels, 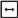 Angiogenesis, 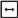 Reepithelialisation
Sigal et al. [66]	7 days	Abdominal surgery	30	14.7 g intravenous Arg	Isonitrogenous solution	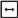 Lymphocyte proliferation, 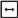 NO
Langkamp-Henken et al. [60]	4 weeks	Elderly people with pressure ulcers	33	0, 8.5 or 17 g Arg	Not supplemented	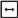 Lymphocyte proliferation, 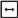 NO, 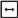 IL-2

Arg: arginine; Cu: copper; Gln: glutamine; HBM: β-hydroxy-β-methylbutyrate; IGF-1: insulin-like growth factor; NO: nitric oxide; P: phosphorous; RME: resting metabolic expenditure; Zn: zinc; ↑: increases; 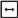 does not increase or decrease.

#### Collagen Deposition (Hydroxyproline Content)

Several studies have demonstrated that supplementation with arginine increases collagen deposition and, therefore, enhances wound-breaking strength. The wound-breaking strength is the force needed to disrupt a wound [67]. Barbul et al. [59] observed this improvement in a randomized, controlled trial (RCT) in 36 healthy and non-smoking humans by supplementing their diet with 24.8 g of free arginine as arginine hydrochloride and 17 g of free arginine as arginine aspartate per day for 2 weeks. Hydroxyproline content was assessed as an index of the synthesis and deposition of new collagen in a polytetrafluoroethylene tube inserted in the wound site. An enhanced collagen deposition at 137 and 74% was noted in the arginine hydrochloride (*p* = 0.028; 23.85 ± 2.16 nmol/cm) and arginine aspartate (*p* = 0.028, 17.57 ± 2.16 nmol/cm) groups, respectively, following a significant difference observed in the controlled group (*p* < 0.001; 10.1 ± 2.32 nmol/cm in controls). These results were confirmed later by Nussbaum [64], who carried out a similar trial in 45 healthy elderly people, randomly supplemented or not with 17 g of arginine per day for 14 days. An improvement was also observed in collagen synthesis through the hydroxyproline deposition (17.4 ± 2 nmol/cm; *p* < 0.02) and T-cell-mediated immune function.

Arginine also influences the nitrogen balance. Nevertheless, this balance improvement has been reported in many, but not in all studies. In a randomized double-blind controlled study by Debats et al. [65], specific parameters related to wound healing were measured after supplementing 30 g of arginine (*n* = 16) or placebo (*n* = 19) for 10 days. Angiogenesis, assessed as the number of vessels per high power field significantly increased on day 10 (8.9 ± 3.1 in the arginine group vs. 8 ± 2.8 in the placebo group, *p* < 0.001). Meanwhile, the increment in re-epithelialization failed to reach significance (85 ± 7.1% in the arginine group vs. 81 ± 8.5% in the placebo group, *p* > 0.05).

In a double-blind RCT conducted and involving 30 adults for 7 days by Sigal et al. [66], the enhancement of lymphocyte proliferation was not observed (*p* > 0.05) after intravenous supplementation with 14.7 g of arginine compared to controls, treated with Travasol 10% (an isonitrogenous mix of amino acids). The nitrogen balance measured in the supplemented group (−8.8 g/day) was comparable to the control group (−9.2 g/day, *p* > 0.05).

Langkamp-Henken et al. [60], also conducted an RCT on 33 elderly patients supplemented with different amounts of arginine (0, 8.5, or 17 g) for 4 weeks. These amounts of arginine used represented approximately 2.2 and 4.5% of an 1800 kcal intake, respectively. The mean daily intake of the patients in this study ranged between 1713 and 2474 kcal with an additional 2.4 and 3.3 g of arginine. After the treatment, no increase in lymphocyte proliferation was observed between groups, while NO increased, although not statistically significant (*p* > 0.05).

**Figure 8:**
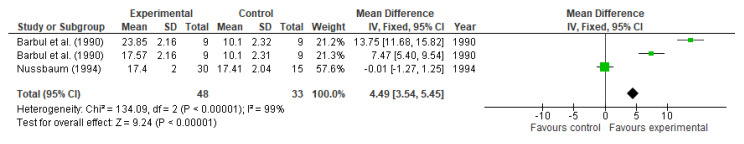
Hydroxyproline content: fixed-effects meta-analysis and forest plot from studies providing supplementation of arginine.

Effects of arginine supplementation on collagen deposition, measured by hydroxyproline content were reported in 2 studies. The study conducted by Barbul et al. [59] reported two results due to two different amounts of supplementation given (17 and 24.8 g arginine). Compared to control, arginine supplementation significantly enhanced hydroxyproline content (MD: 4.49, 95% CI: 3.54, 5.45, *p* < 0.00001, Figure 8). There was a high heterogeneity (I^2^ = 99%) among studies. However, there was a lack of significance between the intervention and control group in the Nussbaum study [64] (MD: −0.01, 95% CI: −1.27, 1.25).

### 3.4. Glutamine (Gln)

Glutamine has been shown to influence several parameters involved in wound healing. This behavior was confirmed by many of the randomized controlled trials that will be reported below, where patients with a total, partial parenteral or early enteral nutrition were supplemented with alanine-glutamine dipeptide, in a concentration ranging from 0.2 to 0.5 g Ala-Gln/kg/day for up to 14 days, as summarized in Table 3. Ala-Gln dipeptide is usually used instead of free Gln, due to its heat stability and its rapid hydrolyzation to free amino acids in plasma [68].

**Table 3:** Studies evaluating the effect of glutamine supplementation on wound healing and reported outcomes.

Study	Duration	Patient Population	*n*	Dosage		Control Group	Outcome
				Gln (g/kg BW/day)	Gln Dip (g/kg BW/day Ala-Gln)		
Oʼriordain et al. [69]	5 days	Surgery	22	0.18	0.27	Isonitrogenous solution	↑ T-cell lymphocytes, 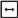 IL-2, 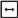 IL-6, 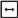 TNFα
De Beaux et al. [70]	7 days	Critical illness	14	0.22	0.33	Isonitrogenous solution	↑ Lymphocytes, ↓ IL-8, 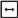 IL-6
Morlion et al. [71]	5 days	Surgery	28	0.2	0.3	Isonitrogenous solution	↑ NO, ↓ LOS and patient mortality, ↑ Mood and general well-being
Jacobi et al. [72]	7 days	Surgery	34	0.27	0.4	Isonitrogenous solution	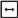 IL-10, ↑ Wound healing
Jiang et al. [73]	7 days	Abdominal surgery	60	0.34	0.5	Isonitrogenous solution	↑ NO, ↓ LOS
Powell-Tuck et al. [74]	4–16.5 days	Mixed	168	0.26	0.38	Isonitrogenous solution	↓ LOS and patient mortality
Mertes et al. [75]	6 days	Abdominal surgery	37	0.34	0.5	Isonitrogenous solution	↑ NO, ↓ LOS, 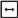 IL-6
Karwowska et al. [76]	10 days	Surgery	30	0.2	0.3	Isonitrogenous solution	↑ IgA and IgG, ↑ T-cell lymphocytes, ↓ LOS
Neri et al. [77]	>7 days	Surgery	33	0.2	0.3	Isonitrogenous solution	↓ LOS
Wischmeyer et al. [78]	>7 days	Critical illness	31	0.57	0.85	Isonitrogenous solution	↓ CRP
Goeters et al. [61]	>9 days	Surgery	144	0.2	0.3	Isonitrogenous solution	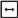 NO, ↓ Patient mortality
Lin et al. [79]	6 days	Abdominal surgery	48	0.28	0.417	Isonitrogenous solution	↑ NO, ↓ IL-6, ↑ T-cell lymphocytes
Ockenga et al. [80]	>7 days	Critical illness	28	0.2	0.3	Isonitrogenous solution	↑ Lymphocytes, ↓ LOS, ↓ CRP
Fuentes-Orozco et al. [81]	10 days	Surgery	10	0.27	0.4	Isonitrogenous solution	↑ NO, ↓ Infectious complications, ↑ Lymphocytes CD4 CD8
Xian-Li et al. [62]	14 days	Critical illness	69	0.27	0.4	Isonitrogenous solution	↓ LOS and patient mortality
Zhou et al. [82]	12 days	Critical illness	30	0.34	0.5	Isonitrogenous solution	↑ Wound healing, ↓ Intestinal permeability
Quan et al. [83]	7 days	Abdominal surgery	20	0.53	0.78	Not specified	↓ Intestinal permeability
Kłek et al. [84]	12 days	Surgery	105	0.27	0.4	Isonitrogenous solution	↑ Lymphocytes, ↓ LOS
Lin et al. [85]	6 days	Abdominal surgery	48	0.28	0.417	Isonitrogenous solution	↑ NO, ↓ IL-6
Yao et al. [86]	5 days	Surgery	40	0.34	0.5	Isonitrogenous solution	↓ LOS, ↑ CD14
Déchelotte et al. [87]	5 days	Surgery	143	0.3	0.45	Isonitrogenous solution	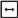 LOS, ↑ NO, ↓ Intestinal permeability
Şahin et al. [88]	10.5 ± 3.6 days	Critical illness	40	0.3	0.45	Isonitrogenous solution	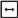 T-cell lymphocytes, ↓ LOS
Cai et al. [89]	14 days	Critical illness	110	0.19	0.29	Isonitrogenous solution	↑ T-cell lymphocytes, ↓ CRP
Duška et al. [90]	13 days	Critical illness	30	0.2	0.3	Isonitrogenous solution	↑ NO
Estívariz et al. [91]	7 days	Surgery	63	0.34	0.5	Isonitrogenous solution	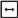 T-cell Lymphocytes, ↓ Nosocomial infections
Dong et al. [92]	6 days	Abdominal surgery	40	0.35	0.5	Isonitrogenous solution	↑ T-cell lymphocytes, ↓ TNFα, ↓ IL-2R
Fuentes-Orozco et al. [93]	10 days	Critical illness	44	0.27	0.4	Isonitrogenous solution	↑ T-cell lymphocytes, ↑ IgA, ↑ NO, ↓ CRP, ↑ IL-10, ↓ IL-6
Yeh et al. [94]	7 days	Surgery	70	0.2	0.29	Isonitrogenous solution	↓ CRP, ↓ LOS, 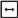 Patient mortality
Asprer et al. [95]	5 days preoperatively	Abdominal surgery	34	0.2	0.3	Isonitrogenous solution	↑ Lymphocytes
Engel et al. [63]	3 days	Surgery	58	0.5	0.74	Isonitrogenous solution	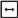 T-cell lymphocytes
Fan et al. [96]	7 days	Abdominal surgery	40	0.13	0.2	Isonitrogenous solution	↓ LOS and infectious complications
Quan et al. [97]	4 days	Abdominal surgery	20	0.35	0.5	Normal saline	↑ NO, ↓ IL-6
Andrews et al. [98]	Up to 7 days	Mixed	502	0.2	0.3	Isonitrogenous solution	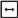 Patient mortality
Çekmen et al. [99]	>5 days	Mixed	30	0.35	0.5	Isonitrogenous solution	↓ CRP, ↓ LOS and patient mortality
Grau et al. [100]	5–9 days	Mixed	127	0.35	0.5	Isonitrogenous solution	↓ Nosocomial infections
Lu et al. [101]	7 days	Surgery	50	0.3	0.45	Isonitrogenous solution	↑ NO, ↓ IL-6, ↓ CRP, ↓ Infectious complications
Wernerman et al. [102]	7 days	Mixed	413	0.28	0.42	Normal saline	↓ LOS and patient mortality
Xu et al. [103]	12 days	Critical illness	80	Unknown	Unknown	Isonitrogenous solution	↓ TNFα
Richard et al. [104]	3 days pre, 4 days postoperatively	Surgery	22	0.53	0.78	Not supplemented	↓ LOS and patient mortality, ↓ CRP

Ala: alanine; BW: body weight; CRP: C-reactive protein; Gln: glutamine; Gln dip: glutamine dipeptide; IgA: immunoglobulin A; IL-2R: interleukin-2 receptor; IL-6: interleukin-6; LOS: length of stay; NO: nitric oxide; TNFα: tumor necrosis factor α; ↑: increases; ↓: decreases; 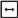 does not increase or decrease.

#### 3.4.1. Nitrogen Balance

In two double-blind RCT performed in 48 patients supplemented with 0.28 g/kg/day Gln for 6 days, by Lin et al. [79,85], higher nitrogen balance, although not significant (−3.2 ± 1.6 vs. −6.5 ± 2.7 g N, *p* > 0.05) was observed after Gln treatment. Cumulative nitrogen balance was higher in Glu-supplemented patients with lower illness severity, assessed with APACHE II scores (acute physiology score + age points + chronic health points), but this was not observed when severity was higher (APACHE II score > 6). However, when compared with controls, the reducing effect was not reported, showing that the improving effect on cumulative nitrogen balance is not due to the reduction of muscle protein breakdown, but an enhanced protein synthesis.

In contrast, a higher cumulative nitrogen balance, adjusted to standard body surface area, was not noted (−193 ± 50 vs. −198 ± 77 g N, *p* > 0.05) in a prospective double-blind RCT conducted by Duška et al. [90], who supplemented 30 patients suffering from critical illnesses with 0.2 g/kg/day Gln for 13 days, and compared the results against the placebo group, treated with isonitrogenous nutrition. Similar results were reported by Mertes et al. [75] in a double-blind RCT on 37 patients undergoing major abdominal surgery for 5 days (−14.1 ± 9.1 vs. −31.7 ± 11.4 g N, *p* < 0.05). Jiang et al. [73] also noted a rise of the cumulative nitrogen balance (144.3 ± 145.6 vs. −5.1 ± 162.7 mg/kg, *p* = 0.0004) in a double-blind RCT in 60 patients undergoing abdominal surgery, supplemented with 0.34 g/kg/day or an isonitrogenous solution for 7 days.

On the contrary, in a double-blind RCT performed by Morlion et al. [71] in 28 patients undergoing surgery, supplemented with 0.2 g/kg/day Gln for 5 days, the increase in cumulative nitrogen balance was also observed (−7.9 ± 3.6 vs. −233.0 ± 2.6, *p* < 0.01).

**Figure 9:**
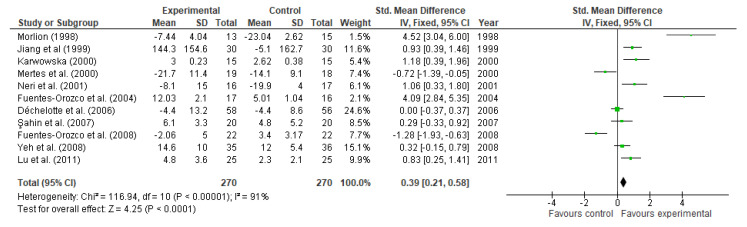
Nitrogen balance: fixed-effects meta-analysis and forest plot from studies providing supplementation of glutamine.

Eleven studies reported data on nitrogen balance. Compared to control, glutamine supplementation significantly increased nitrogen balance (MD: 0.39, 95% CI: 0.21, 0.58, *p* < 0.0001, Figure 9). There was a high heterogeneity (I^2^ = 91%) among studies. Nevertheless, results reported by 3 studies lacked from significance. These studies are Déchelotte et al. [87] (MD: 0.00, 95% CI: −0.37, 0.37), Şahin et al. [88] (MD: 0.29, 95% CI: −0.33, 0.92), and Yeh et al. [94] (MD: 0.32, 95% CI: −0.15, 0.79).

#### 3.4.2. Wound Healing Time

Two trials [72,82] measured the effect of glutamine supplementation on wound healing. Zhou et al. [82] performed a double-blind RCT and noticed a lower infection rate (13 vs. 26%), although this difference failed to reach significance (*p* > 0.05). Also, it was noted a significant reduction of the wound healing time in the study group (32.1 ± 3.3 days) compared to the control group (36.6 ± 6.6 days, *p* < 0.012) after supplementing 30 patients suffering from severe burns with 0.34 g/kg/day Gln or an isonitrogenous solution, for 12 days. This data is in agreement with the RCT performed by Jacobi et al. [72] where, after supplementing 34 patients with 0.27 g/kg/day Gln for 7 days, observed a significant increase in wound healing (*p* < 0.05), explained in part by the incidence of wound complications post-surgery.

#### 3.4.3. Length of Hospital Stay (LOS) and Patient mortality

The decrease in LOS was reported by many studies. Neri et al. [77] studied the effect of 0.2 g/kg/day Gln in 33 patients supplemented for at least 7 days. In this trial, the study group spent 11.5 ± 2 days hospitalized, versus the control group, which spent 15 ± 3 days (*p* < 0.05). Meanwhile, in the prospective double-blind RTC carried out by Yao et al. [86] in 40 people for 5 days, the study group spent 10.6 ± 1.2 days in the hospital, while the non-supplemented group spent 11.7 ± 2 days (*p* = 0.03).

These data are in agreement with Fan et al. [96], who performed an RCT in 40 patients undergoing surgery and observed a trend to a reduction both in LOS (22.3 ± 2.1 vs. 24.9 ± 1.7 days, *p* = 0.32) and infectious complications after supplementation with 0.13 g/kg/day Gln, although these data failed to reach significance. The reduction in LOS and mortality was also observed by Wischmeyer et al. [78] in a double-blind RCT in 31 patients with severe burns, supplemented with 0.57 g/kg/day L-Gln for at least 7 days via parenteral feeding. However, in this case, there was no significant difference seen in the LOS (31 ± 10.1 vs. 30 ± 9.3, *p* > 0.05) nor mortality (1 vs. 4 deaths, *p* = 0.19).

Goeters et al. [61] also noticed a patient mortality decline within 6 months (11/33 vs. 21/35 deaths, *p* < 0.05), resulting in a 66.7% increase of survival of patients treated for ≥ 9 days, compared to 40% in controls, in an unblinded parallel multicentre RCT in 144 critically ill patients supplemented with 0.2 g/kg/day Gln.

Despite the lack of statistical significance, Çekmen et al. [99] also observed a trend to the reduction in LOS (19.3 ± 12.7 vs. 27.5 ± 12.1 days, *p* = 0.36) and patient mortality (20 vs. 40%, *p* = 0.42) in a double-blind RCT in 30 critically ill patients supplemented with 0.35 g/kg/day Gln.

Karwowska et al. [76] studied the effect of glutamine supplementation and observed a decrease in LOS; from 15.1 ± 3 days to 12.5 ± 1.2 days (*p* = 0.005) in the supplemented group. The reduction in LOS was also seen in a double-blind RCT carried out by Powell-Tuck et al. [74] in 168 patients, supplemented with 20 g Gln as part of the nitrogen source of parenteral nutrition or standard feeds, for 7 days. The study group spent an average of 15 fewer days in hospital, compared with the control group (46 ± 10.7 vs. 30 ± 7.2 days, *p* < 0.03). The latter study failed to demonstrate a decrease in morbidity and mortality in the study group, probably due to the younger age of the patients (48 ± 17 years old vs. 80 ± 19 years old). Nevertheless, this limitation was avoided in a study by Xian-Li et al. [62] in a non-blinded RCT in 69 patients suffering from critical illnesses for 14 days, and it was seen, not only a reduction in LOS (25.3 ± 7.6 vs. 39.1 ± 10.6 days, *p* < 0.01), but also in patient mortality (0 vs. 43.5%, *p* < 0.01) after supplementing 0.2 g/kg/day Gln. The same results were obtained by Wernerman et al. [102] in a double-blind RCT, observing a reduction of patient mortality in ICU (8/11 vs. 14/20, *p* < 0.05) after supplementing 413 patients, in ICU, with 0.28 g/kg/day Gln for 7 days.

On the other hand, Ockenga et al. [80] reported a significant reduction in LOS in a double-blind RCT in 28 patients suffering from acute pancreatitis supplemented with 0.2 g/kg/day Gln for at least 7 days. In this trial, the LOS was an average of 4 days shorter in the study group (21 days) compared to the control group (25 days, *p* < 0.05) despite the small sample studied. In the trial conducted by Mertes et al. [75] a decrease in hospital stay was also noted (12.8 ± 2.6 vs. 17.5 ± 6.4 days, *p* < 0.05) compared to the control group. Similar results were observed by Jiang et al. [73] who noted a decrease in 4 days of LOS (12.5 ± 5.1 vs. 16.4 ± 7.1 days, *p* = 0.02). Morlion et al. [71] not only noted the reduction in LOS (15.5 ± 0.7 vs. 21.7± 2.8 days, *p* < 0.05), but there were also evident improvements both in mood and general well-being in the supplemented patients.

On the contrary, in a prospective double-blind RCT performed by Déchelotte et al. [87] in 114 patients with critical illnesses treated with 0.33 g/kg/day Gln, no significant differences in LOS nor patient mortality were observed (1.9 vs. 3.8% deaths, *p* > 0.05), although a reduction of the incidence of infectious complications (39 vs. 64%, *p* < 0.02) was noted. The decrease of nosocomial infections, such as urinary tract infections (2.3 vs. 16.9%, *p* = 0.03) and pneumonia (8 vs. 29%, *p* = 0.02) were also observed by Grau et al. [100]. This trial studied the effect of 0.35 g/kg/day Gln in a multicentre, prospective, double-blind, RCT in 502 patients admitted to the UCI. In another double-blind RCT performed by Estívariz et al. [91] in 63 people undergoing surgery and in ICU, patients were supplemented with 0.34 g/kg/day Gln and a lessening of nosocomial infections was observed (13 vs. 36 cases, *p* < 0.03). Kłek et al. [84] also reported a decrease in postoperative complications (23.3 vs. 36.6%, *p* < 0.05) and LOS (14.8 vs. 16.4 days, *p* < 0.05) in an RCT performed in 69 patients undergoing surgery supplemented with 0.27 g/kg/day Gln for 12 days.

On the contrary, a double-blind RCT conducted by Andrews et al. [98], patients supplemented with 0.2 g/kg/day Gln for at least 5 days, neither showed a significant improvement in the incidence of infections nor patient mortality compared to the control group (*p* > 0.05).

**Figure 10:**
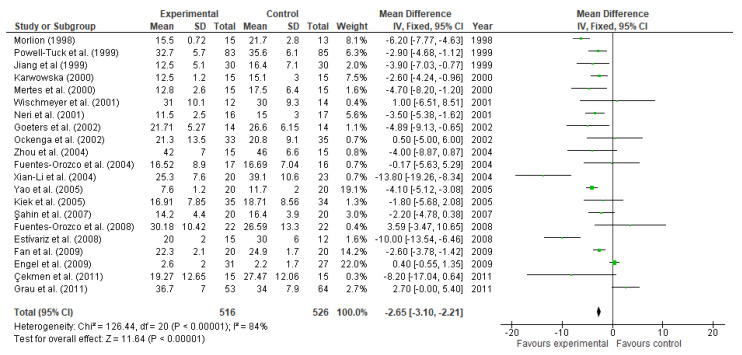
Length of hospital stay: fixed-effects meta-analysis and forest plot from studies providing supplementation of glutamine.

The effect of glutamine on LOS was reported by 21 studies involving 1042 participants. According to the meta-analysis, glutamine supplementation has shown to significantly decrease the days of hospitalization (MD: −2.65, 95% CI: −3.10, −2.21, *p* < 0.00001, Figure 10). Exhibiting a high heterogeneity among studies (I^2^ = 84%). Nevertheless, not all the studies showed a significant effect on this parameter. This is the case of the following studies: Wischmeyer et al. [78] (MD: 1.00, 95% CI: −6.51, 8.51), Ockenga et al. [80] (MD: 0.50, 95% CI: −5.00, 6.00), Zhou et al. [82] (MD: −4.00, 95% CI: −8.87, 0.87), Fuentes-Orozco et al. [81] (MD: −0.17, 95% CI: −5.63, 5.29), Kiek et al. [84] (MD: −1.80, 95% CI: −5.68, 2.08), Şahin et al. [88] (MD: −2.20, 95% CI: −4.78, 0.38), Fuentes-Orozco et al. [93] (MD: 3.59, 95% CI: −3.47, 10.65), Engel et al. [63] (MD: 0.40, 95% CI: −0.55, 1.35), Çekmen et al. [99] (MD: −8.20, 95% CI: −17.04, 0.64).

**Figure 11:**
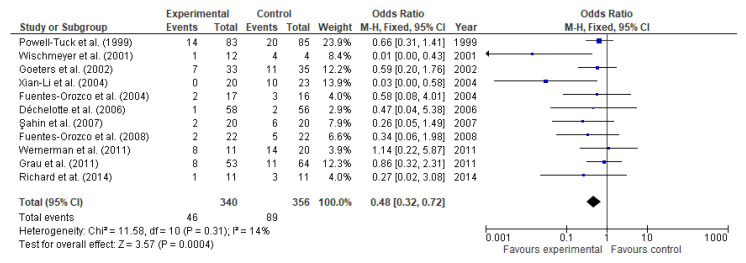
Patient mortality: fixed-effects meta-analysis and forest plot from studies providing supplementation of glutamine.

Overall data from 11 studies involving 696 participants (340 and 356 in the study and control group respectively) were evaluated. Patient mortality occurred less frequently in the glutamine-supplemented group than in controls (46 (13.53%) vs. (89 (25%) participants). The odds ratio was 0.48 (95% CI: 0.32, 0.72, *p* = 0.0004, Figure 11), and heterogeneity among studies was 14%. The number needed to treat was 10. In other words, 10 patients would need to receive glutamine supplementation to prevent an additional fatality.

#### 3.4.4. Lactulose/Mannitol Ratio

Zhou et al. [82] observed a decrease in the intestinal permeability in the study group (0.021 ± 0.006 vs. 0.025 ± 0.007 L/M ratio, *p* = 0.115). These data are in agreement with those by Jiang et al. [73] who also noted a decrease in intestinal permeability, assessed by lactulose/mannitol excretion rate ratio (L/M ratio) (0.097 ± 0.063 vs. 0.132 ± 0.081 L/M ratio, *p* = 0.02). Similar results were reported by Xu et al. [103] who also noted the reduction of L/M (*p* < 0.05).

**Figure 12:**
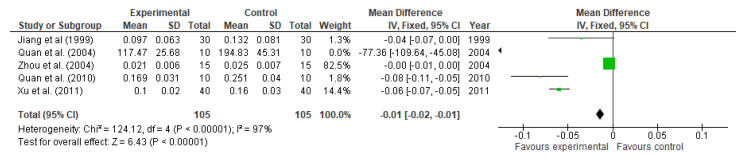
Lactulose/mannitol ratio: fixed-effects meta-analysis and forest plot from studies providing supplementation of glutamine.

Five studies involving 210 participants reported data on intestinal permeability, assessed by lactulose/mannitol ratio. L/M significantly decreased in those participants supplemented with glutamine (MD: −0.01, 95% CI: −0.02, −0.01, *p* < 0.00001, Figure 12). The heterogeneity given by I^2^ was 97%.

#### 3.4.5. C-Reactive Protein

Çekmen et al. [99] observed a trend to the reduction in CRP (57.65 ± 41.81 vs. 82.87 ± 69.41 mg/L, *p* = 0.32) compared to the control group. Nevertheless, these results did not reach significance.

A double-blind RCT performed by Lu et al. [101] on 50 patients, suffering from gastrointestinal cancer and undergoing surgery, supplemented with 0.3 g/kg/day Gln; reported a significant reduction in CRP serum level (16.7 ± 11.8 vs. 35.2 ± 24.8 mg/dL, *p* = 0.013). Besides, in the control group, there were noted 4 cases of infectious complications while none were observed in the study group (*p* = 0.037). The reduction in CRP (*p* < 0.01) was also observed by Wischmeyer et al. [78] in a double-blind RCT in 31 patients with severe burns, supplemented with 0.57 g/kg/day L-Gln for at least 7 days via parenteral feeding. Ockenga et al. [80], as well as Dong et al. [92] also reported a significant decrease in CRP (30 ± 42 vs. 34 ± 51, *p* < 0.01), (69 ± 19 vs. 99 ± 44, *p* = 0.01)

Yeh et al. [94] noted the CRP lessening (*p* = 0.012) in an RCT in 70 patients undergoing surgery for 7 days. Another RCT performed by Cai et al. [89] suggested a pronounced decrease of CRP (25.8 ± 4.9 vs. 15.6 ± 4.2 mg/dL, *p* = 0.001) after performing an RCT supplementing 110 patients suffering from a critical illness with 0.19 g/kg/day Gln for 14 days. Besides, it was also suggested a decrease in the illness severity, assessed by APACHE II scores (10.35 ± 4.35 vs. 17.75 ± 4.46, *p* = 0.001). Richard et al. [104] supplemented patients before and after surgery and also observed a significant decrease in CRP (44 ± 35 vs. 69 ± 19 mg/L, *p* = 0.028)

**Figure 13:**
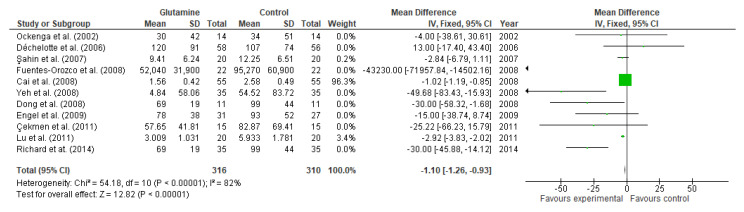
C-reactive protein: fixed-effects meta-analysis and forest plot from studies providing supplementation of glutamine.

Eleven studies reported the effect of glutamine supplementation on CRP levels. Although, individually, 5 studies data did not reach statistical significance [63,80,87,88,99], the overall effect showed the reduction of CRP levels after supplementation (MD: −1.10, 95% CI: −1.26, −0.93, *p* < 0.00001, Figure 13). The heterogeneity among studies was 82%.

#### 3.4.6. Cytokines

Quan et al. [97] observed a significant reduction of IL-6 (6.95 ± 5.08 vs. 12.88 ± 3.85 ng/L, *p* < 0.05) when 20 patients undergoing abdominal surgery were supplemented with 0.28 g/kg/day Gln for 4 days in a multicentre double-blind RCT. The reduction in IL-6 was also reported by Lu et al. [101] (19.2 ± 9.8 vs. 34.7 ± 18.7 pg/mL, *p* = 0.01).

On the other hand, Xu et al. [103] noted a reduction in TNFα levels (*p* = 0.01) in an RCT in 80 patients supplemented with glutamine administered via early enteral nutrition.

O’Riordain et al. [69] performed a double-blind RCT in 22 patients undergoing surgery and supplemented with 0.18 g/kg/day Gln in the form of glycyl-glutamine, for 5 days. This trial measured the IL-6 and TNFα levels. Nevertheless, the data failed to reach significance (*p* = 0.27 and *p* > 0.05, respectively). The lack of significance regarding the production of IL-6 was also observed in a double-blind RCT performed by De Beaux et al. [70] in 14 patients suffering from critical illnesses.

**Figure 14:**
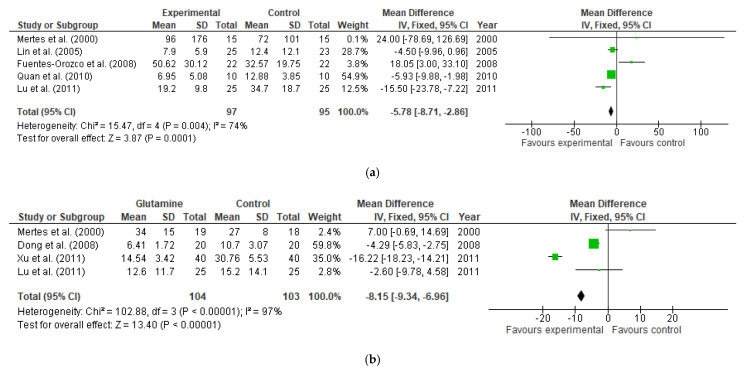
(**a**) IL-6 levels: fixed-effects meta-analysis and forest plot from studies providing supplementation of glutamine (**b**) TNFα levels: fixed-effects meta-analysis and forest plot from studies providing supplementation of glutamine.

Five and four studies reported data on IL-6 and TNFα levels, respectively. Both parameters showed a significant reduction after glutamine supplementation for IL-6 levels (MD: −5.78, 95% CI: −8.71, −2.86, I^2^ = 74, *p* = 0.0001) and for TNFα levels (MD: −8.15, 95% CI: −9.34, −6.96, I^2^ = 97, *p* < 0.00001, Figure 14a,b). This occurred despite the lack of significance shown by 2 different studies in both parameters. For IL-6 levels this was the case of Mertes et al. [75] (MD: 24.00, 95% CI: −78.69, 126.69) and Lin et al. [85] (MD: −4.5, 95% CI: −9.96, 0.96) while for TNFα levels they were Mertes et al. [75] (MD: 7.00, 95% CI: −0.69, 14.69) and Lu et al. [101] MD: −2.60, 95% CI: −9.78, 4.58).

#### 3.4.7. T-Cell Lymphocytes

Karwowska et al. [76] performed an RTC and studied the effect of glutamine in the immune system by the increase of T-cells, particularly, CD4 and CD8 lymphocytes, IgA, IgG in well-nourished male patients undergoing abdominal surgery, supplemented with 0.202 g/kg/day Gln. Significant increases in the total counts of lymphocytes (*p* = 0.005), CD4 lymphocytes (*p* = 0.005), CD8 lymphocytes (*p* = 0.01), IgA (*p* = 0.005) and IgG (*p* = 0.02) were observed. Kłek et al. [84] also reported an increase in T-cell lymphocytes in the group supplemented with Gln. Nevertheless, this measurement did not reach significance (*p* > 0.05).

Asprer et al. [95] also noticed an increase in T-cell lymphocytes count (*p* = 0.049) in a prospective RCT performed in 34 patients supplemented with 0.3 g/kg/day Gln for 5 days before surgery. This data is in agreement with Ockenga et al. [80], who also reported an increase in lymphocytes (1.7 ± 0.7 vs. 1.5 ± 0.4, *p* < 0.01). Similar results were reported by Cai et al. [89] (1.91 ± 0.33 vs. 1.35 ± 0.25 × 10^9^/L, *p* = 0.001) and Dong et al. [92] (*p* < 0.01).

On the contrary, O’Riordain et al. [69] reported a significant increase in the synthesis of T-cell DNA, measured with the tritiated thymidine index, compared to the control group (*p* < 0.05).

In a double-blind RCT carried out by Fuentes-Orozco et al. [81] for 10 days, on 33 patients with secondary peritonitis, supplemented with 0.26 g/kg/day Gln, it was also suggested an increase in T-cells levels, including, their subpopulations, CD4 and CD8. However, no significant difference was reached (*p* > 0.05).

Regarding the effect of glutamine supplementation on T-cell lymphocytes, there is contradictory evidence in the literature.

**Figure 15:**
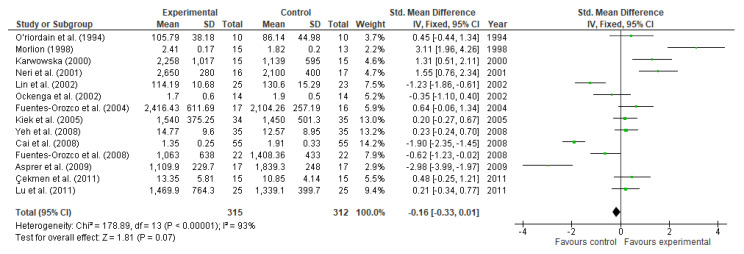
T-cell lymphocytes: fixed-effects meta-analysis and forest plot from studies providing supplementation of glutamine.

However, fourteen studies involving 540 participants (315 and 312 in the experimental and control group, respectively) showed that glutamine supplementation might increase T-cell lymphocytes levels. Nevertheless, the data analysis showed a lack of significance in the measured parameter (MD: −0.16, 95% CI: −0.33, 0.01, I^2^ = 93, *p* = 0.07, Figure 15).

## 4. Discussion

### 4.1. Arginine

Based on the findings of the systematic review, arginine supplementation resulted in greater collagen formation assessed by hydroxyproline level (*p* < 0.00001). The deposition of collagen could in part be enhanced by T-cell-mediated immune function since they recruit and activate fibroblasts which play a key role in wound repair [105].

The effect of arginine supplementation on T-cell lymphocytes and nitrogen balance was reported in some studies. Nevertheless, its beneficial effect was not always observed [66]. A reason that could explain this outcome may be the lack of additional calories administered either parentally or enterally, which attenuates or even eliminates the pharmaceutical effect of arginine [18,19]. This occurs because around 40% of arginine is catabolized in a single pass in the small intestine, by the type II arginase, and to a much lesser extent, by NO synthase [106]. Indeed, Castillo et al. [107] stated that only 0.34% of the arginine intake absorbed in the splanchnic bed is used to synthesize NO, contributing to 16% of the daily production of NO. Attempts to meet energy requirements were done in most trials, with a minimum of 120 kcal/day in the trial conducted by Debats et al. [65] and a maximum of 2474 kcal/day in the study performed by Langkamp-Henken et al. [60]. Some studies did not show a significant increase or decrease in lymphocyte proliferation and nitrogen balance, respectively [60]. The explanation for this could be the timing of the measurements. Since the elimination of half-life of an oral load of arginine is about 80 min [108].

### 4.2. Glutamine

Regarding glutamine supplementation, the greater cumulative nitrogen balance noted in some of these studies explains the use of glutamine by the body as a substrate for the synthesis of NO. Improved nitrogen retention is associated with a shorter length of hospital stay, and high levels of IL-6 are associated with infections and mortality. The lower production of proinflammatory cytokines might also be explained due to the decrease in intestinal permeability [48]. Therefore, an increase in the nitrogen balance and decline of IL-6 may explain why in many studies, there was a reduction in the length of hospitalization and patient mortality observed [61]. However, the decrease in mortality was not always significant in this review, hence not fully agreeing with the review conducted by Bollhalder et al. [53].

In a prospective double-blind RCT performed by Déchelotte et al. [87] in 114 patients with critical illnesses, no significant differences in LOS nor patient mortality were observed (1.9 vs. 3.8% deaths, *p* > 0.05), and even if there was an improvement in the nitrogen balance, this variation did not reach significance (−2.44 ± 8.6 vs. −4.4 ± 13.2, *p* > 0.05). In this case, the benefits of supplementation may have been due to the decrease in intestinal permeability [40,44,46], resulting thus, in the reduction of the incidence of infectious complications (39 vs. 64%, *p* < 0.02). The decrease of the infectious complications (4 vs. 12 cases, *p* < 0.005) could be possibly influenced by the increase of the nitrogen balance (12 ± 2 vs. 5 ± 1 g N, *p* < 0.05), as well as the levels of IgA (335.7 ± 31.44 vs. 357.81 ± 83.61 mg/dL, *p* = 0.029) and IgG between intervention and the control group [81].

Şahin et al. [88] reported a lessening in CRP in both groups, although the decrease was more pronounced in the study group (−38 vs. 18.6%, *p* = 0.00 and *p* = 0.01, respectively). However, these values were still higher than usual and a reason for this could be the presence of fewer leukocytes, and inflammation. In this trial, there was a reduction in LOS (14.2 ± 4.4 vs. 16.4 ± 3.9 days, *p* > 0.05) and complication rates (10 vs. 40%, *p* < 0.05) which may be due to an increase in CD4 and CD8 lymphocytes, although the latter failed to reach significance (*p* > 0.05). The increase in T-cell lymphocytes was also reported in other studies [80,83,87]. Nevertheless, in line with the latter, these measurements did not reach significance (*p* > 0.05).

Fuentes-Orozco et al. [93] reported a significant increase of nitrogen balance (*p* = 0.04), total lymphocytes count (*p* = 0.04), CD4 lymphocytes (*p* = 0.03), CD8 lymphocytes (*p* = 0.03), IgA (*p* = 0.01) and IL-10 (*p* = 0.02), an anti-inflammatory cytokine, were observed, along with a significant decrease of CRP (0.005) and IL-6 (*p* = 0.03). These data might explain the decrease in the incidence of infectious complications in the supplemented group (68.4%) when compared to the control group (31.6%, *p* = 0.03), as well as the mortality (9 vs. 22.7%), although the latter failed to reach significance (*p* = 0.20).

According to a review conducted by Novak et al. [54], the minimum concentration of glutamine to obtain positive clinical outcomes is 0.2 g/kg/day Gln, corresponding to 0.303 g/kg/day Ala-Gln dipeptide. Nevertheless, this amount could be raised to 20 g/day Gln according to Wischmeyer et al. [78], Heyland et al. [109] and García-De-Lorenzo [110]. Regarding Gln toxicity, Garlick [111] suggested that doses as high as 50–60 g/day Gln for several weeks were safe and showed no adverse effect. The use of a lower amount of glutamine could in part explain the lack of significance in the reduction of cytokines levels in some studies [69]. In the study by Engel et al. [63], dose requirements according to the previous studies [54,78,109,110] were met. In this case, a reason to explain these results may be the short time used for supplementation (3 days) rather than the dose used.

The form of supplementation used might also affect the outcome, even if the minimum amount of glutamine is given. This could be explained since glutamine in the form of L-Gln presents much lower stability than Ala-Gln dipeptide [78]. In addition, the effect of glutamine may also vary depending on whether it is given postoperatively and parenterally, and whether it is given preoperatively [95,104] and enterally [103], respectively. The observed beneficial effect on L/M ratio and TNFα levels was more pronounced when glutamine was administered by the enteral route. However, since the authors do not specify the amount of the administered glutamine, it is difficult to determine whether this observation is dose dependent or is due to the method of administration being the enteral route.

The shortest study [63] supplemented patients for just 3 days with 0.5 g/kg/day Gln in a double-blind RCT. In this trial, no significant differences were found in total lymphocytes count (*p* > 0.05), IL-6 levels (*p* < 0.05), LOS (2.6 ± 2.0 vs. 2.0 ± 1.7, *p* = 0.44), CRP (*p* = 0.72), IL-8 (*p* > 0.05) or TNFα (*p* > 0.05). This might suggest that a minimum length of 5 days is needed to achieve an effect in at least one of the parameters related to healing [69].

Besides, according to Morlion et al. [71], glutamine supplementation may also have a positive effect on the patient’s mood and general well-being. A reason for this could be the role of Gln as a neurotransmitter [112,113] as its depletion has been shown to cause mood disorders such as depression [113,114,115].

Therefore, it would be useful to measure this parameter in future studies to corroborate this effect.

## 5. Limitations of the Review

In the case of arginine, five studies were included in the meta-analysis. However, it was not possible to assess the effect of arginine supplementation on nitrogen balance and T-cell lymphocytes due to the lack of studies reporting these data. Besides, the studies had relatively small sample sizes. Therefore, more studies are required in this area of research. Besides, the high heterogeneity of the studies of both amino acids might have also affected the results of the meta-analysis.

## 6. Conclusions

This systematic review and meta-analysis have demonstrated that supplementation with either arginine and glutamine can positively influence wound healing or parameters related to healing including LOS and mortality. The effect of arginine supplementation was significant in relation to hydroxyproline content (*p* < 0.00001), while glutamine supplementation had significant effect on nitrogen balance (*p* < 0.0001), patient mortality (*p* = 0.0004), L/M ratio (*p* < 0.00001), LOS (*p* < 0.00001), CRP (*p* < 0.00001), IL-6 levels (*p* = 0.0001) and TNFα levels (*p* < 0.00001). However, the effect of glutamine supplementation on T-cell lymphocytes failed to reach significance (*p* = 0.07).

## Figures and Tables

**Table 1:** Search terms and search strategy.

Patient/Population	Intervention	Outcome	Study Designs	Combining Search Terms
**Patients**	Amino acid		Randomized controlled trial	
Patients undergoing Surgery OR patients with Pressure Ulcers OR Patients with Critical Illness	Amino Acid OR Arginine OR Glutamine OR Nutrition	Inflammation OR Healing OR Wound OR Cytokines OR Interleukin OR Hospital Stay OR C-reactive protein	Clinical trial OR Randomised controlled trial OR controlled clinical trial	Column 1 AND Column 2 AND Column 3 AND Column 4
